# Acute Retroperitoneal Hemorrhage Due to Multi-Vessel Aneurysmal Bleeding As the Initial Manifestation of Polyarteritis Nodosa in a Previously Healthy Female

**DOI:** 10.7759/cureus.45718

**Published:** 2023-09-21

**Authors:** Marco A Albornoz, Lexi Branca, Andrew Samoyedny

**Affiliations:** 1 Rheumatology, Main Line Health System/Riddle Memorial Hospital, Media, USA; 2 Rheumatology Medical Scribe, Main Line Health System/Riddle Memoral Hospital, Media, USA; 3 Radiology, University of Pennsylvania, Philadelphia, USA

**Keywords:** wunderlich's syndrome, pseudoaneurysm, aneurysm, spontaneous abdominal bleeding, polyarteritis nodosa

## Abstract

Polyarteritis nodosa (PAN) is a heterogeneous disease characterized by constitutional symptoms, multi-organ dysfunction, and a subacute to chronic mode of onset. We describe a rarely encountered manifestation of PAN in a previously healthy 64-year-old woman who developed acute intra-abdominal bleeding due to multi-vessel, vasculitis-induced retroperitoneal aneurysmal ruptures, followed by the spontaneous cessation of abdominal pain and intra-abdominal bleeding within 24 hours after hospitalization, in the absence of immunosuppressive treatment. Aneurysms regressed and eventually normalized coincident with a moderate-term course of oral corticosteroids and six months of monthly intravenous cyclophosphamide. Our report reveals that patients with life-threatening PAN may present acutely with unexpected historical, physical examination and laboratory features not commensurate with the gravity of the clinical findings. We also highlight that long-term survival lasting greater than 11 years can occur when the correct diagnosis and appropriate treatment are introduced early in the course of the illness.

## Introduction

The presence of spontaneous intra-abdominal bleeding as the de novo manifestation of polyarteritis nodosa (PAN) in a previously healthy person is rare, as 93% of non-viral induced PAN patients present with constitutional symptoms and evidence of multi-organ dysfunction [1]. We describe the case of a previously healthy 64-year-old female who was hospitalized with acute, severe atraumatic PAN-induced abdominal pain and retroperitoneal bleeding due to aneurysmal ruptures, followed by abrupt multi-vessel healing in the absence of symptom-directed treatment. Contextually unexpected clinical features, pathophysiologic and outcome correlates are also reviewed.

## Case presentation

A 64-year-old Caucasian female with a history of indolent primary Raynaud’s phenomenon, two decades of asymptomatic coronary artery disease, and hypertension presented to the emergency room with the abrupt onset of severe atraumatic abdominal pain, moderate nausea, and low-volume non-bloody emesis. The patient was in excellent health until the time of admission with no prior constitutional symptoms and without remarkable social and family histories. On admission, the patient was in distress due to severe generalized, non-radiating abdominal pain. She was alert and oriented, and not chronically ill-appearing. She was afebrile, normotensive, in normal sinus rhythm with a pulse of 80, and a pulse oximetry reading of 97% on room air. The physical examination immediately after admission to the emergency room revealed a soft abdomen with normal bowel sounds and negligible, non-localized abdominal discomfort without rebound or bilateral flank tenderness. The remainder of the examination was normal. Complete blood count, comprehensive metabolic panel, prothrombin time (PT)/partial prothrombin time (PTT), and urinalysis were normal and remained normal throughout the entire seven-day hospital stay. Subsequent blood cultures, and serologies for HIV, hepatitis C, and B, antinuclear antibody (ANA), and anti-neutrophil cytoplasmic antibody (ANCA) were negative. Erythrocyte sedimentation rate and C reactive protein obtained on the day of admission were elevated at 61 mm/hr and 58 mg/L, respectively. Echocardiography and chest x-ray were normal.

**Figure 1:**
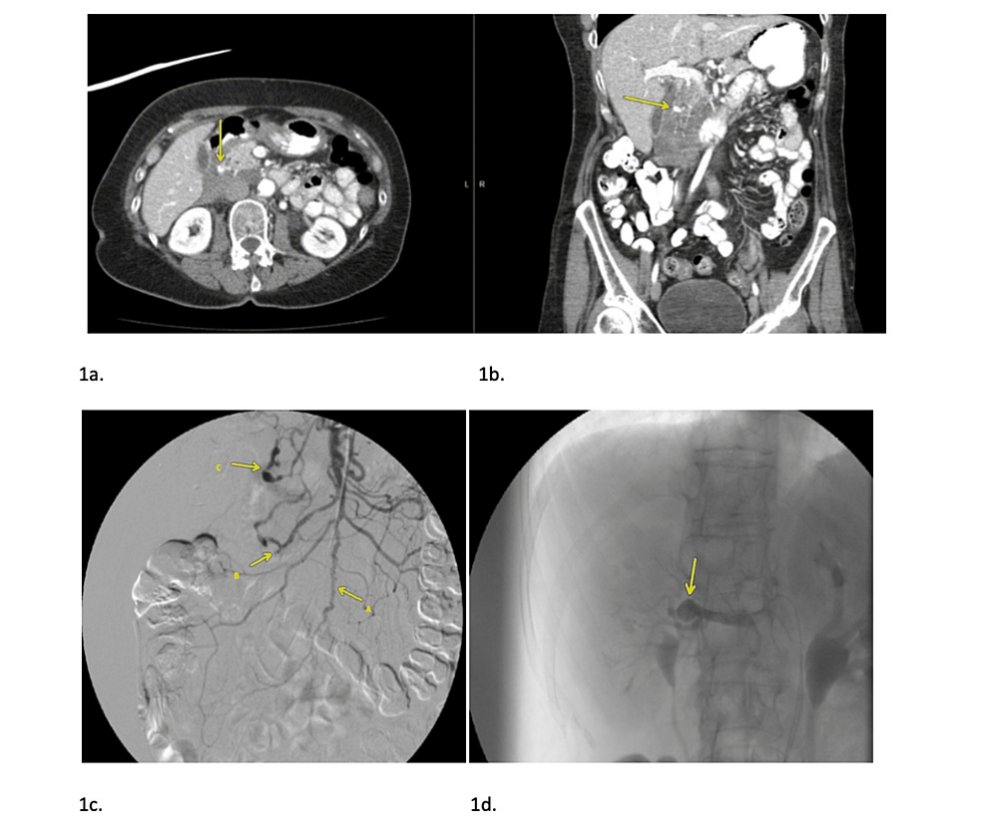
1a - Axial and coronal reformatted images from a contrast enhanced CT examination day 1 of admission demonstrating peripancreatic hemorrhage (arrow). 1b - Rounded foci of high density material (arrow) highly suspicious for peripancreatic pseudoaneurysm. 1c - Superior mesenteric artery (SMA) arteriogram from day 1 demonstrating diffuse irregularity of a jejunal branch of the SMA (Arrow A) as well as multiple stenoses and luminal irregularity of SMA branches (arrows B and C). 1d - Arteriogram of the right renal artery demonstrating a 2.7 cm aneurysm of the renal artery at the renal hilum (arrow).

**Figure 2:**
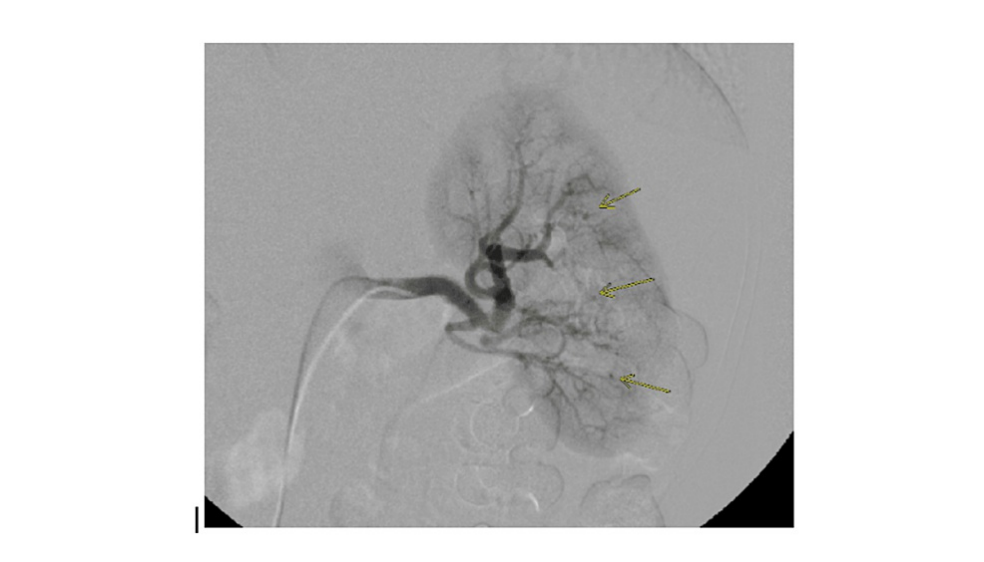
Arteriogram of the left kidney from day 1 of admission demonstrating multiple rounded foci of contrast pooling (arrows), suggesting small pseudoaneurysms.

Intravenous (IV) contrast-enhanced computed tomographic angiography of the abdomen and pelvis on hospital day one revealed pooling of contrast in the retroperitoneum, and aneurysmal rupture as the likely arterial source of acute hemorrhage. Selective arteriography of the superior and inferior mesenteric arteries on day one revealed diffuse irregularity along with multifocal stenosis and dilatation of the pancreaticoduodenal arcade and jejunal branches. Additional findings include high-grade stenosis at the origin of the celiac artery and irregularity of the hepatic artery as well as a right hilar renal aneurysm (Figure 1) and left renal artery pseudoaneurysms (Figure 2).

**Figure 3:**
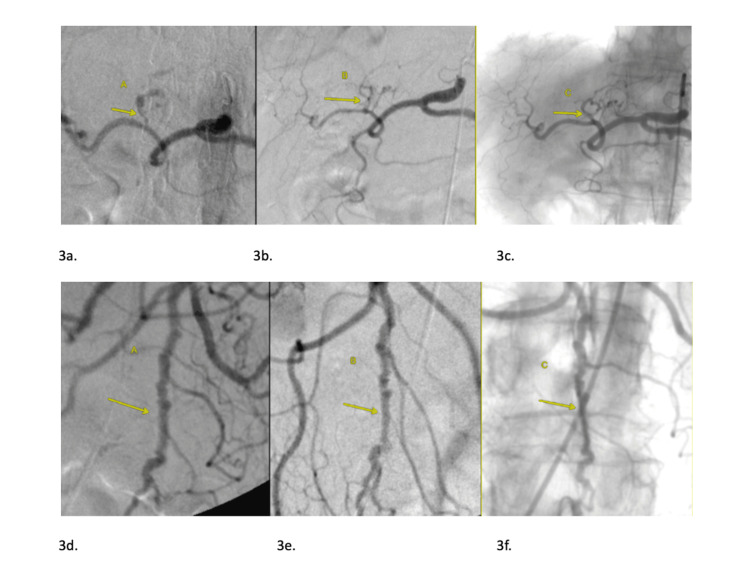
3a. Celiac arteriogram from day 1 of admission demonstrates luminal irregularity and stenosis of left hepatic artery (arrow A). 3b. Follow-up arteriogram 5 months post hospitalization reveals moderate vessel contour improvement (arrow B). 3c. Arteriogram 26 months post hospitalization (arrow C) shows normalization of vessel caliber and luminal irregularities. 3d. Superior mesenteric artery arteriogram from hospital day 1 reveals luminal irregularity of a jejunal branch (arrow A). 3e. Follow-up arteriogram from 5 months after treatment reveals moderate improvement (arrow B). 3f. Arteriogram 26 months post treatment reveals normalization of affected vessels (arrow C).

On day three of hospitalization, the patient was treated with 1g IV methylprednisolone on three consecutive days, before being discharged on prednisone 60mg daily, followed by a steroid taper and a six-dose course of IV cyclophosphamide (600mg/m2) administered monthly. At five months after discharge, while taking prednisone 10mg daily, surveillance angiography revealed 90% diminution of vasculitic findings, followed by total resolution 26 months after the start of treatment (Figure 3). The patient has been in excellent health for 11 years post hospitalization.

## Discussion

The great majority of patients with intra-abdominal PAN present in a subacute or chronic timeline with organ-threatening clinical findings that traditionally require immunosuppressive therapy, often in conjunction with arterial catheter-based radiologic interventions and/or surgery. PAN involving the gastrointestinal (GI) tract conveys a grave risk of mortality [1]. In our patient, despite the high risk of developing a catastrophic outcome, the following highly infrequent findings were encountered: a) abrupt onset of life-threatening symptoms in a previously healthy person as the first manifestation of the disease; b) normal vital signs, and relatively benign physical examination findings throughout the hospital stay; c) normal complete blood count, comprehensive metabolic panel and urinalysis; d) acute cessation of intra-abdominal bleeding in the absence of conventional symptom-directed therapies; and, e) The 11-year follow-up interval of excellent patient health.

The clinical manifestations and pathogenesis of PAN are diverse and may present alone or in combination with other diseases and syndromes [1,2]. Although multiple variants of PAN have been identified, in broad terms, the vast majority of patients with PAN are divided into hepatitis B-predominant viral and non-viral subsets. In contrast to the viral-associated PAN, whose destructive effects are predominantly immune complex-mediated, the idiopathic, or classical, non-viral PAN phenotype lacks this immunological profile. Rather, intramural necrotizing damage preferentially involving the medium-sized arteries with sparing of arterioles, venules, and capillaries, is the pathological process that ultimately leads to the downstream sequelae of ischemia, stenosis, and aneurysm formation that is generally seen in both groups.

Our patient’s idiopathic PAN phenotype is also seen in Wunderlich’s syndrome, a rare condition known by the triad of flank pain, flank mass, and hypovolemic shock or Lenk’s triad, which is characterized by spontaneous bleeding into the perirenal space, and most commonly caused by renal neoplasias in 61% of patients, followed in frequency by PAN at 15% [3,4]. In these two PAN subtypes, aneurysmal rupture-mediated blood loss represents the shared pathogenic mechanism. The precise frequency and pathophysiology of spontaneous aneurysm healing in PAN is unknown. Our review of the literature identified 81 reports [5-39] of PAN patients who presented solely with acute disease limited to the intra-abdominal cavity, 43 of which had Wunderlich’s syndrome [5-17]. Therefore, when managing patients with spontaneous, atraumatic intra-abdominal hemorrhage in the absence of a mass or bleeding diathesis, PAN should be placed high on the list of diseases to exclude. In this patient, despite the presence of simultaneous widespread vasculitis activity, and/or aneurysm and pseudoaneurysm formation involving the hepatic, renal, superior, and inferior mesenteric arteries, and select tributaries, the not uncommon PAN scenario of absent clinical clues by way of symptoms, signs or laboratory data indicating organ-specific involvement was encountered [3]. Other case reports have shown the correlation between angiographic resolution of intra-abdominal aneurysms and with resolution of symptoms following treatment with steroid therapy and cyclophosphamide [40]. This patient exhibited complete arterial healing 26 months following the initial presentation of acute retroperitoneal hemorrhage, which, to our knowledge, is the longest period of imaging-confirmed resolution described in the PAN literature.

## Conclusions

Our case reveals that intra-abdominal PAN can present acutely, and do so with life-threatening consequences. However, when appropriate diagnostic and therapeutic interventions are instituted early, angiographic abnormalities can normalize and long-term, morbidity-free survival is attainable.

## References

[1] Pagnoux C, Seror R, Henegar C (2010). Clinical features and outcomes in 348 patients with polyarteritis nodosa: a systematic retrospective study of patients diagnosed between 1963 and 2005 and entered into the French Vasculitis Study Group Database. Arthritis Rheum.

[2] Zizic TM, Classen JN, Stevens MB (1982). Acute abdominal complications of systemic lupus erythematosus and polyarteritis nodosa. Am J Med.

[3] Karadag O, Jayne DJ (2018). Polyarteritis nodosa revisited: a review of historical approaches, subphenotypes and a research agenda. Clin Exp Rheumatol.

[4] Zhang JQ, Fielding JR, Zou KH (2002). Etiology of spontaneous perirenal hemorrhage: a meta-analysis. J Urol.

[5] Beirão P, Teixeira L, Pereira P, Coelho ML (2017). Wunderlich's syndrome as a manifestation of polyarteritis nodosa. BMJ Case Rep.

[6] Katabathina VS, Katre R, Prasad SR, Surabhi VR, Shanbhogue AK, Sunnapwar A (2011). Wunderlich syndrome: cross-sectional imaging review. J Comput Assist Tomogr.

[7] Lee SH, Yun SJ (2017). Polyarteritis nodosa presenting as bilateral Wunderlich syndrome: rare cause of flank pain in a young woman. Am J Emerg Med.

[8] Venkatramani V, Banerji JS (2014). Spontaneous perinephric hemorrhage (Wunderlich syndrome) secondary to polyarteritis nodosa: Computed tomography and angiographic findings. Indian J Urol.

[9] Agarwal A, Bansal M, Pandey R, Swaminathan S (2012). Bilateral subcapsular and perinephric hemorrhage as the initial presentation of polyarteritis nodosa. Intern Med.

[10] Kambayashi Y, Iseri K, Yamamoto Y, Abe M, Wada Y, Yanai R, Honda H (2022). Bilateral renal subcapsular hematoma caused by polyarteritis nodosa: a case report. CEN Case Rep.

[11] Cheng MM, Yen CS, Li CM, Chien CC, Kan W (2014). Spontaneous bilateral perirenal hemorrhage following prolonged fever: an uncommon presentation of polyarteritis nodosa. Clin Nephrol.

[12] Bindi M, Quartieri F, Scatena P, Filardo FP, Castiglioni MG (2001). Spontaneous perirenal hemorrhage in patients with polyarteritis nodosa (Article in Italian). Recenti Prog Med.

[13] Johansen M, Thomsen GH (1999). Spontaneous rupture of the kidney with perirenal hemorrhage in undiagnosed polyarteritis nodosa (Article in Danish). Ugeskr Laeger.

[14] Unverdi S, Altay M, Duranay M, Krbas I, Demirci S, Yuksel E (2009). Polyarteritis nodosa presenting with splenic infarction, bilateral renal infarction, and hematoma. South Med J.

[15] Zapzalka DM, Thompson HA, Borowsky SS, Coleman-Steenson CC, Mahowald ML, O'Connell KJ (2000). Polyarteritis nodosa presenting as spontaneous bilateral perinephric hemorrhage: management with selective arterial embolization. J Urol.

[16] Nandwani GM, Musker MP, Chaplin BJ, El Madhoun I, Akbani H (2013). Spontaneous perirenal haemorrhage in polyarteritis nodosa. J Coll Physicians Surg Pak.

[17] Paparella MT, Eusebi L, Gangai I, Bartelli F, Guglielmi G (2021). Wunderlich syndrome: a rare case in a young woman. Acta Biomed.

[18] Georgiou C, Krokidis M, Elworthy N, Dimopoulos S (2016). Spontaneous bilateral renal aneurysm rupture secondary to Polyarteritis Nodosa in a patient with chronic myelomonocytic leukaemia: a case report study. Int J Surg Case Rep.

[19] Minardi D, Dessì-Fulgheri P, Sarzani R (2003). Massive spontaneous perirenal hematoma and accelerated hypertension in a patient with polyarteritis nodosa. Urol Int.

[20] Waisayarat J, Niyasom C, Vilaiyuk S, Molagool S (2022). Polyarteritis nodosa with cytomegalovirus enteritis and jejunoileal perforation: report of a case with a literature review. Vasc Health Risk Manag.

[21] Seifarth FG, Ibrahim S, Spalding SJ, Reid JR (2014). Intestinal obstruction secondary to infantile polyarteritis nodosa. Afr J Paediatr Surg.

[22] Bulbuloglu E, Kantarceken B, Yuksel M, Ciralik H, Sahinkanat T, Kale IT (2006). An unusual presentation of polyarteritis nodosa: a case report. West Indian Med J.

[23] Cengiz N, Demir S, Parmaksız G, Temiz AK, Noyan A (2012). Polyarteritis nodosa: a case presenting with renal mass. Eur J Pediatr.

[24] Levine Sm, Hellmann Db, Stone Jh (2002). Gastrointestinal involvement in polyarteritis nodosa (1986-2000): presentation and outcomes in 24 patients. Am J Med.

[25] Vaidya GN, Khorasani-Zadeh A, John S (2014). Polyarteritis nodosa presenting as profuse gastrointestinal bleeding. BMJ Case Rep.

[26] El Madhoun I, Warnock NG, Roy A, Jones CH (2009). Bilateral renal hemorrhage due to polyarteritis nodosa wrongly attributed to blunt trauma. Nat Rev Urol.

[27] Hiraike Y, Kodaira M, Sano M (2013). Polyarteritis nodosa diagnosed by surgically resected jejunal necrosis following acute abdomen. World J Gastroenterol.

[28] Tanakaya K, Konaga E, Takeuchi H (2001). Penetrating colon ulcer of polyarteritis nodosa: report of a case. Dis Colon Rectum.

[29] Cengız C, Pampal K, Doğan S, Bulut S, Boyacioğlu S (2010). Acute abdomen due to intestinal ischemia as an initial presentation of polyarteritis nodosa. Turk J Gastroenterol.

[30] Hayakawa A, Nakajima T, Satoh K (2013). Case report: a case of polyarteritis nodosa with small intestinal perforation and renal aneurysm rupture (Article in Japanese). Nihon Naika Gakkai Zasshi.

[31] Gómez-Luque I, Alconchel F, Ciria R (2016). Spontaneous liver rupture as first sign of polyarteritis nodosa. World J Hepatol.

[32] Peddi P, Kalavakunta JK, Annakula M, Armstrong JR (2010). An unusual complication of polyarteritis nodosa with massive retroperitoneal hemorrhage: a case report. Int Arch Med.

[33] Kapur SV, Oswal JS (2021). Acute pancreatitis as a presenting feature in a child with systemic polyarteritis nodosa. Indian J Pathol Microbiol.

[34] Corbitt M, Kurtkoti J, Rashid M, Nigam S (2019). Polyarteritis nodosa presenting atypically as appendicitis and pyelonephritis in a single patient. BMJ Case Rep.

[35] Lin LC, Stone J, Singh S, Hsieh TC, Subramony R (2022). Atraumatic bilateral renal subcapsular urinomas in a young, healthy female. J Emerg Med.

[36] Chattopadhyay A (2001). Intestinal perforation due to polyarteritis nodosa. Indian J Pediatr.

[37] Mukhopadhyay P, Rathi M, Kohli HS, Jha V, Gupta KL, Sakhuja V (2012). Polyarteritis nodosa presenting with spontaneous perirenal hematoma. Indian J Nephrol.

[38] Tsai HC, Liao HT, Tsai CY (2020). Polyarteritis nodosa with intra-hepatic arterial haemorrhage. Liver Int.

[39] Kitzing B, O'Toole S, Waugh A, Clayton J, McGill N, Allman KC (2009). Hepatobiliary scintigraphy in vasculitis of the gallbladder as a manifestation of polyarteritis nodosa: a case report. Cases J.

[40] Harada M, Yoshida H, Ikeda H (1999). Polyarthritis nodosa with mesenteric aneurysms demonstrated by angiography: report of a case and successful treatment of the patient with prednisolone and cyclophosphamide. J Gastroenterol.

